# Technological impacts of antibiotic residues in animal-derived fermented foods: Mechanisms, detection challenges, regulatory gaps, and mitigation strategies

**DOI:** 10.14202/vetworld.2026.2991-3007

**Published:** 2026-07-17

**Authors:** Mbarga Manga Joseph Arsene, Bassa Zacharie Carime, Parfait Kezimana, Ibrahim Khelifi, Anyutoulou Kitio Linda Davares, Elena Vasilyeva, Nadezhda Sachivkina, Maria Molchanova, Natallia Zhabo, Marina Avdonina, Ntolo Bomba Arly Thérèse

**Affiliations:** 1 Department of Microbiology, V.S. Kiktenko Medical Institute, Peoples' Friendship University of Russia named after Patrice Lumumba (RUDN University), Moscow, Russian Federation.; 2 Department of Agrobiotechnology, Agrarian Institute, Peoples' Friendship University of Russia named after Patrice Lumumba (RUDN University), Moscow, Russian Federation.; 3 Department of Foreign Languages, Institute of Medicine, Peoples' Friendship University of Russia named after Patrice Lumumba (RUDN University), Moscow, Russian Federation.; 4 Department of Linguistics and Intercultural Communication, Moscow State Linguistic University, Moscow, Russian Federation.

**Keywords:** Animal-derived foods, antimicrobial resistance, antibiotic residues, biosensors, detection methods, fermented foods, food safety, mitigation strategies

## INTRODUCTION

Antibiotic residues (ARs) in foods of animal origin pose a multifaceted challenge at the intersection of public health, food technology, and veterinary practice, raising concerns ranging from consumer safety to the sustainability of traditional food systems [1, 2]. In livestock production, antibiotics are widely administered for therapeutic, prophylactic, and growth-promoting purposes; however, their misuse and overuse often result in residual compounds persisting in milk, meat, and fish [3–5]. When inadequately controlled, these residues enter the food chain, creating risks for both consumers and food systems [1, 2, 6–10].

Numerous studies have reported high prevalence rates of AR contamination, with certain regions exhibiting particularly elevated levels due to weak regulatory enforcement and inadequate compliance with withdrawal periods [2, 11]. Although the public health implications of ARs, particularly their contribution to antimicrobial resistance (AMR), are well established, their effects on food-processing technologies remain comparatively underexplored. A key technological concern is the interference of ARs with microbial fermentation, a process central to the production of many foods [12, 13]. Fermentation relies on microbial metabolism to enhance sensory, nutritional, and safety attributes [14–18]. Even at low concentrations, antibiotics can inhibit or eliminate beneficial microorganisms, resulting in fermentation failures, quality defects, and economic losses [12, 14, 19, 20]. This risk is particularly evident in dairy products such as cheese and yogurt, fermented meat products such as salami, and traditional fish products such as fish sauce [13, 21].

Mechanistically, different antibiotic classes disrupt microbial pathways involved in cell wall synthesis and protein translation, directly impairing lactic acid bacteria (LAB), yeasts, and halophilic microbiota. These disruptions can lead to delayed acidification, impaired enzymatic activity, and fermentation failure, ultimately compromising product quality, safety, and shelf life [6, 12].

Beyond technological disruption, ARs carry important public health implications. Chronic dietary exposure to low levels of antibiotics contributes to the emergence and dissemination of AMR, which is now recognized as a global health crisis [1, 15, 22, 23]. AMR undermines the effectiveness of antibiotics in both human and veterinary medicine [24, 25]. In addition, ARs can trigger hypersensitivity reactions, allergic responses, and, in some cases, carcinogenic effects [26, 27].

In addition to microbial inhibition, AR interference may promote pathogen survival and biogenic amine accumulation in fermented products, further increasing food safety risks. Despite these significant technological implications, most previous studies have focused primarily on public health and regulatory aspects, with limited integration of microbiological, pharmacological, and food-processing perspectives. Furthermore, differences in susceptibility among industrial, artisanal, and traditional fermentation systems remain insufficiently characterized, representing an important area requiring further investigation.

Despite the existence of international regulatory frameworks, compliance remains challenging, particularly in resource-limited settings. Codex Alimentarius and the European Union have established maximum residue limits (MRLs), but effective enforcement depends on sensitive detection systems and producer awareness [11, 28–31]. Although advanced analytical methods such as liquid chromatography–mass spectrometry (LC-MS) and enzyme-linked immunosorbent assay (ELISA) provide high sensitivity and specificity, their cost and technical requirements limit their adoption in many low-income regions [19, 21, 32–34]. The problem also affects small-scale and traditional producers, for whom fermentation practices constitute an important component of cultural heritage [14, 15, 35, 36]. Consequently, AR contamination not only compromises product safety and quality but also threatens the preservation of traditional food-processing practices [11, 20, 37].

Addressing AR-related risks requires an integrated approach that combines scientific innovation, regulatory enforcement, and stakeholder engagement [2, 11, 29, 30, 38, 39]. Promising strategies include developing residue-tolerant starter cultures that maintain fermentation activity under low antibiotic pressure [21, 31, 37] and adopting alternative husbandry practices, such as improved hygiene and vaccination programs, to reduce antibiotic dependence [1, 4, 30]. Equally important are education and awareness programs targeting farmers, processors, and consumers to promote responsible practices and strengthen compliance [3, 11, 28, 30, 39].

Although substantial progress has been made in understanding the occurrence of ARs and their role in AMR, current knowledge regarding their technological consequences in fermentation-based food systems remains fragmented. Most available studies have focused predominantly on toxicological concerns, AMR, and regulatory issues, whereas comparatively little attention has been devoted to the mechanisms through which ARs influence fermentation performance, microbial ecology, and product quality. Furthermore, available evidence is scattered across different food matrixes, and comparative analyses among dairy, meat, and aquatic fermentation systems are limited. Existing studies rarely integrate microbiological, pharmacological, technological, and regulatory perspectives into a unified framework. In addition, differences in susceptibility among industrial, artisanal, and traditional fermentation systems have not been comprehensively evaluated. The absence of standardized thresholds for fermentation inhibition, limited information regarding the long-term effects of ARs on food microbiomes, and insufficient assessment of emerging monitoring technologies represent major knowledge gaps that hinder the development of effective mitigation strategies and harmonized regulatory approaches.

Therefore, this review aims to provide a comprehensive and integrative analysis of the technological risks associated with ARs in foods of animal origin, with particular emphasis on their disruptive effects on microbial fermentation processes. Specifically, the review examines the occurrence of ARs across different food categories and the factors contributing to their persistence, including pharmacokinetic behavior and processing stability [6]. It further analyzes the mechanisms through which different antibiotic classes interfere with fermentation processes and links these effects to measurable technological outcomes, including acidification rate, proteolysis, flavor development, and product quality. In addition, the review critically evaluates current detection and monitoring approaches, highlighting both technological advancements and practical limitations encountered in resource-limited settings. Finally, it identifies key research gaps and proposes a structured mitigation framework that integrates preventive measures, monitoring strategies, and technological interventions, thereby addressing a critical yet underexplored dimension of ARs in food systems.

**Figure 1:**
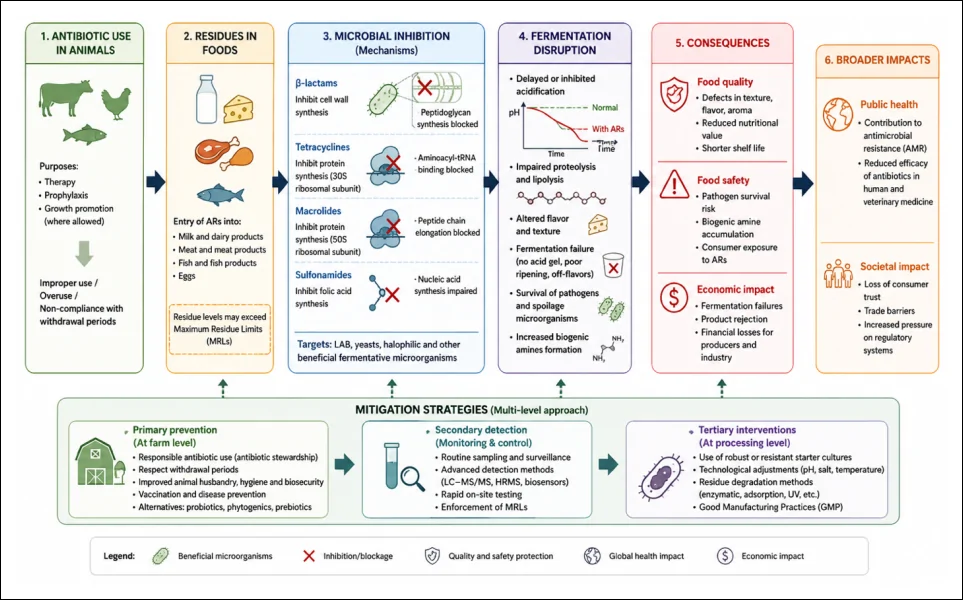
Conceptual framework illustrating the pathways and technological impacts of antibiotic residues (ARs) in animal-derived fermented foods. The figure depicts the progression from antibiotic use in animal production to residue contamination of food matrixes, subsequent microbial inhibition, disruption of fermentation processes, and the resulting consequences for food quality, food safety, economic performance, and public health. In addition, the framework incorporates potential mitigation strategies that can be implemented at different stages of the production chain [4, 21, 30, 39].

A conceptual framework illustrating the pathways and technological impacts of ARs in fermentation systems is presented in Figure 1[4, 21, 30, 39].

## REVIEW METHODOLOGY

This review was conducted using a structured narrative approach to synthesize current knowledge regarding ARs in foods of animal origin and their technological implications. Relevant literature was retrieved from major scientific databases, including PubMed, Scopus, Web of Science, and Google Scholar. The search strategy combined keywords such as “antibiotic residues,” “fermented foods,” “lactic acid bacteria,” “food safety,” “antimicrobial resistance,” and “detection methods.”

The selection criteria focused primarily on peer-reviewed articles published within the last 10 years, with particular emphasis on studies investigating the occurrence of residues, their technological effects on fermentation processes, analytical detection methods, and mitigation strategies. Seminal and highly cited earlier studies were also included, where appropriate, to provide historical background and mechanistic insights.

Studies were included if they provided quantitative or qualitative data on ARs in animal-derived foods, experimental evidence describing their effects on microbial fermentation, or information on analytical approaches for residue detection and monitoring. Articles dealing exclusively with clinical or environmental aspects without direct relevance to food systems were excluded.

Although this review followed a structured approach for literature selection and synthesis, certain limitations should be acknowledged. Variations in study design, analytical methodologies, sampling procedures, and regional surveillance capacities may affect the comparability of reported findings. In addition, differences in regulatory frameworks and residue-monitoring practices among countries may contribute to heterogeneity in the available evidence.

Therefore, the findings presented in this review should be interpreted in light of these methodological and geographical limitations. Nevertheless, the broad inclusion of studies from different regions and production systems provides a comprehensive overview of the current state of knowledge regarding the occurrence, technological impacts, detection, and mitigation of ARs in foods of animal origin.

## OCCURRENCE OF ARS IN FOODS OF ANIMAL ORIGIN

The occurrence of ARs in foods of animal origin has emerged as a major concern for both public health and food safety. These residues primarily result from the administration of antibiotics in livestock production for therapeutic, prophylactic, and, in some cases, growth-promoting purposes. When these drugs are misused or when mandatory withdrawal periods are not observed before slaughter or milk collection, residual compounds may persist in food products such as meat, milk, eggs, and fish [4, 31]. This issue is particularly pronounced in intensive production systems and in regions where regulatory frameworks are weak or inadequately enforced [2].

Beyond their mere presence, ARs exhibit considerable variability across geographical regions, food categories, and antibiotic classes, reflecting differences in veterinary practices, regulatory enforcement, production systems, and surveillance capacities. These variations indicate that AR contamination is influenced not only by antibiotic usage patterns but also by disparities in monitoring infrastructure and regulatory compliance. Consequently, contamination levels vary substantially across countries and production sectors, underscoring the need for context-specific control measures and harmonized surveillance programs.

A comparative synthesis of global prevalence patterns of ARs in foods of animal origin is presented in Table 1[3, 5, 9, 22, 40–47].

Numerous studies worldwide have confirmed the presence of ARs in food products, often exceeding established MRLs. For example, in Nigeria, more than 80% of meat samples tested positive for oxytetracycline residues, frequently exceeding safe thresholds [40]. In Pakistan, approximately 40% of milk samples contained tetracyclines and sulfonamides [3], whereas 32% of beef samples in Ethiopia exceeded MRLs for tetracycline residues [5]. Similarly, in Bangladesh, 28% of poultry meat samples were contaminated with ciprofloxacin residues [22].

These findings indicate that contamination is not only widespread but also heterogeneous, with markedly higher prevalence rates observed in low- and middle-income countries, where enforcement of withdrawal periods and veterinary oversight remains limited [2, 11]. In contrast, high-income regions tend to exhibit lower but persistent contamination levels, reflecting stronger surveillance systems but incomplete elimination of residues. These regional disparities underscore the importance of strengthening residue-monitoring programs and harmonizing regulatory practices at the international level.

### Dairy products

The dairy sector has been extensively studied due to the high global consumption of milk and dairy products. In the European Union, approximately 12% of raw milk samples have been reported to contain ARs, with β-lactams and tetracyclines being the most frequently detected compounds [41]. In Cameroon, penicillin residues have been identified in raw milk obtained from local farms [42]. The persistence of residues in milk is largely influenced by pharmacokinetic factors, including drug lipophilicity, protein binding, and excretion pathways, which facilitate accumulation within mammary tissues. Moreover, incomplete compliance with withdrawal periods remains a major driver of contamination.

### Meat and poultry products

Meat products are equally affected by AR contamination. In Brazil, macrolides and quinolones have been detected in poultry meat, with approximately 15% of samples exceeding permissible limits [43]. In Egypt, oxytetracycline residues have been reported in beef and lamb [44], whereas sulfonamide residues were detected in 20% of beef and chicken samples in Ghana [9]. Compared with dairy products, contamination in meat is often associated with prolonged antibiotic retention in tissues, particularly in muscle and liver, and reflects cumulative exposure throughout the animal's lifespan. Intensive production systems further exacerbate this problem through repeated antibiotic administration.

### Fish and aquaculture products

Seafood, particularly aquaculture-derived products, represents an increasingly important source of AR exposure. In Thailand, farmed shrimp were found to contain high concentrations of fluoroquinolones, including enrofloxacin and ciprofloxacin [45]. In Vietnam, chloramphenicol residues detected in fish have resulted in export restrictions [46], whereas oxytetracycline residues were identified in 18% of aquaculture fish samples in Egypt [9]. Aquaculture systems are especially vulnerable because antibiotics are often introduced directly into water, leading to environmental persistence and bioaccumulation in aquatic organisms. This route of contamination differs considerably from that observed in terrestrial livestock systems, highlighting the need for sector-specific monitoring and management strategies.

**Table 1:** Global occurrence of antibiotic residues in foods of animal origin.

**Food category**	**Region/** **Country**	**Antibiotic class detected**	**Prevalence ** **(%)**	**MRL exceedance**	**Detection method**	**Key observations**	**References**
Meat (beef)	Nigeria	Tetracyclines (oxytetracycline)	>80%	High	LC-MS/microbiological assay	Widespread misuse and poor compliance with withdrawal periods	[40]
Milk	Pakistan	Tetracyclines, sulfonamides	~40%	Moderate–High	ELISA/LC-MS	Dairy contamination associated with prophylactic use	[3]
Beef	Ethiopia	Tetracyclines	~32%	High	LC-MS	Weak regulatory monitoring	[5]
Poultry meat	Bangladesh	Fluoroquinolones (ciprofloxacin)	~28%	High	high-performance liquid chromatography (HPLC)/ELISA	Intensive poultry production pressure	[22]
Raw milk	European Union	β-lactams, tetracyclines	~12%	Low–Moderate	LC-MS	Strong surveillance but persistent low-level contamination	[41]
Milk	Cameroon	β-lactams (penicillin)	Detected	Variable	Microbiological assay	High risk for artisanal dairy fermentation	[42]
Poultry meat	Brazil	Macrolides, quinolones	~15%	Moderate	LC-MS	Influence of intensive industrial production	[43]
Beef and lamb	Egypt	Tetracyclines	Significant levels	Moderate–High	ELISA/LC-MS	Inadequate veterinary oversight	[44]
Meat (beef/chicken)	Ghana	Sulfonamides	~20%	Moderate	Microbiological assay	Limited monitoring infrastructure	[9]
Shrimp (aquaculture)	Thailand	Fluoroquinolones	High	High	LC-MS	Excessive antibiotic use in aquaculture systems	[45]
Fish	Vietnam	Chloramphenicol	Detected	High	LC-MS	Trade-related contamination concerns	[46]
Fish (aquaculture)	Egypt	Tetracyclines	~18%	Moderate–High	ELISA	Aquaculture-associated contamination	[9]
Eggs	India	Tetracyclines	~23%	Moderate	ELISA	Poor compliance with withdrawal periods	[47]
Eggs	Ethiopia	Tetracyclines	~15%	Moderate	LC-MS	Emerging contamination concern	[5]

MRL = Maximum residue limit; LC-MS = Liquid chromatography–mass spectrometry; ELISA = Enzyme-linked immunosorbent assay; HPLC = High-performance liquid chromatography. Prevalence values are reported as presented in the original studies. MRL exceedance indicates the extent to which detected antibiotic residues exceeded the applicable regulatory maximum residue limits established by national or international authorities. "Detected" indicates the presence of antibiotic residues without a reported prevalence percentage. "Significant levels" indicates concentrations reported as substantial by the original study but without a specific prevalence estimate.

### Eggs and other animal-derived products

Eggs are also susceptible to AR contamination. In India, 23% of egg samples contained tetracycline residues [47], whereas 15% of egg samples from Ethiopia showed oxytetracycline contamination [5]. Residues in eggs are primarily associated with systemic distribution of antibiotics in laying hens, followed by deposition within the yolk and albumen. This issue is of particular concern because eggs are widely consumed by vulnerable population groups, including children and older adults.

## FACTORS DRIVING RESIDUE PERSISTENCE

Several factors contribute to the persistence of ARs in food systems. Mismanagement of antibiotics, including overdosing and failure to comply with withdrawal periods, remains the primary cause [2]. Inadequate monitoring systems and limited awareness among farmers further exacerbate the problem [11, 48]. In addition, the physicochemical properties of antibiotics strongly influence their persistence. Certain classes, particularly tetracyclines and sulfonamides, exhibit high thermal stability, allowing them to withstand common processing procedures. Although pasteurization may reduce residue concentrations in milk, complete elimination is not achieved, and cooking has limited effects on thermally stable compounds [6, 12].

## CRITICAL SYNTHESIS AND IMPLICATIONS

**Overall, the global distribution of ARs reveals a consistent pattern:** higher contamination levels in regions with limited regulatory enforcement and lower yet persistent levels in highly regulated systems. Importantly, the current body of literature remains largely descriptive, with relatively few comparative analyses across geographical regions and food categories. Furthermore, the absence of standardized risk-ranking systems for antibiotic classes based on their technological and microbiological impacts represents a major gap in both research and policy development.

These findings highlight the importance of addressing ARs at their source through improved antibiotic stewardship, stricter enforcement of withdrawal periods, and strengthened surveillance systems. Although technological innovations, including residue-tolerant starter cultures and alternative farming practices, offer promising opportunities, their successful implementation will require coordinated efforts among governments, regulatory agencies, industry stakeholders, and researchers [19, 21]. Ultimately, effective control of ARs is essential not only for protecting public health but also for maintaining food quality, ensuring technological reliability, and preserving the sustainability of animal-derived food systems [1, 41].

## TECHNOLOGICAL RISKS OF ARS IN FERMENTED ANIMAL-DERIVED FOODS

The presence of ARs in foods of animal origin represents a major technological challenge for fermentation-based food systems. Fermentation is a highly regulated biological process driven by complex microbial consortia, including LAB, yeasts, and, in some cases, halophilic microorganisms, which transform raw substrates into stable, safe, and organoleptically desirable products such as yogurt, cheese, fermented meats, and fish sauces [14–16]. Even trace concentrations of ARs can disrupt this delicate microbial balance, leading to fermentation failure, product defects, and reduced process reliability [12, 31].

From a mechanistic perspective, the technological risks associated with ARs arise from their direct interference with microbial metabolic pathways. Different antibiotic classes exert specific inhibitory effects: β-lactams disrupt cell wall synthesis, tetracyclines inhibit protein synthesis, and sulfonamides interfere with folate metabolism, collectively impairing microbial growth, enzyme production, and stress adaptation. These effects are particularly critical for starter cultures, whose metabolic activities govern acidification, proteolysis, and flavor development [3, 20]. Table 2 summarizes the major classes of antibiotics that may be present as residues in animal-derived raw materials. For each class, the mechanism of action, target microorganisms, impact on fermentation processes, potential product defects, and associated technological risk level are presented [3, 6, 12, 20, 31, 43, 45, 46].

At the core of fermentation processes lies the activity of starter cultures, which ensure controlled acidification and product consistency. ARs interfere with these processes by inhibiting microbial growth and metabolic activity. For example, penicillin residues at concentrations as low as 0.005 mg/L have been shown to delay acidification during yogurt production, demonstrating that even sub-MRL concentrations can significantly affect fermentation performance [31].

Despite these observations, a major limitation of the current literature is the lack of standardized dose-response relationships defining the threshold concentrations at which fermentation inhibition occurs. Although several studies have reported inhibitory effects of ARs at sub-MRL concentrations, available data remain fragmented and inconsistent among antibiotic classes and fermentation systems. For instance, β-lactams have been shown to inhibit LAB at concentrations as low as 0.005 mg/L, whereas comparable thresholds for tetracyclines and sulfonamides remain poorly characterized. This lack of harmonized dose-response information limits the development of predictive models for fermentation failure and represents a critical barrier to risk assessment and regulatory standardization.

**Table 2:** Mechanistic impact of antibiotic residues on fermentation processes in animal-derived foods.

**Antibiotic class**	**Mechanism of action**	**Target microorganisms**	**Fermentation impact**	**Product defects/consequences**	**Technological risk level**	**References**
β-lactams (e.g., penicillin)	Inhibition of cell wall synthesis	LAB	Delayed or inhibited acidification; reduced starter culture growth	Poor curd formation, weak texture, off-flavors, and reduced shelf life	High	[12, 20]
Tetracyclines	Inhibition of protein synthesis (30S ribosome)	LAB, spoilage bacteria	Reduced microbial growth and metabolic activity; incomplete fermentation	Slow acidification, spoilage, and accumulation of undesirable compounds	High	[3, 6]
Sulfonamides	Inhibition of folate synthesis	LAB and other bacteria	Impaired microbial metabolism and enzymatic activity	Reduced fermentation efficiency and unstable product quality	Moderate	[6]
Macrolides	Inhibition of protein synthesis (50S ribosome)	LAB	Suppressed microbial growth and enzyme production	Altered flavor development and incomplete fermentation	Moderate–High	[43]
Fluoroquinolones (e.g., ciprofloxacin and enrofloxacin)	Inhibition of DNA gyrase and topoisomerase IV	LAB, halophilic bacteria	Reduced microbial replication and enzymatic activity	Delayed fermentation and weak flavor development in fish products	High	[45, 46]
Chloramphenicol	Inhibition of protein synthesis (50S ribosome)	Broadspectrum microorganisms (LAB and halophiles)	Severe inhibition of fermentation microbiota	Fermentation failure and loss of characteristic sensory properties	High	[46]
Heat-stable antibiotics (e.g., tetracyclines and sulfonamides)	Persistence during processing	LAB and other fermentation microbiota	Continued inhibition during and after heat treatment	Persistent fermentation disruption despite processing	High	[6, 31]

LAB = Lactic acid bacteria; DNA = Deoxyribonucleic acid.

### Dairy fermentation systems

The dairy industry is particularly vulnerable to AR contamination due to its reliance on LAB-driven fermentation. In cheese production, β-lactam residues impair enzymatic processes involved in curd formation and ripening, resulting in structural defects, off-flavors, and reduced shelf life [12]. Similarly, delayed acidification during yogurt production compromises both texture and microbial safety [20].

In artisanal systems, such as those reported in Cameroon, penicillin-contaminated milk has been shown to reduce both the yield and quality of fermented dairy products [42]. These effects are often more pronounced in artisanal and traditional systems, where fermentation depends on spontaneous microbiota rather than standardized starter cultures, rendering them more susceptible to AR-induced disruption. In contrast, industrial systems may exhibit partial resilience due to controlled inoculation and process standardization, though they are not immune to fermentation failure.

### Meat fermentation systems

Fermented meat products, including sausages and salami, rely on LAB such as *Lactobacillus sakei* and *Pediococcus acidilactici* for acidification, preservation, and flavor development [15]. AR contamination, particularly by tetracyclines, inhibits these microorganisms, leading to incomplete fermentation and increased spoilage risks [6].

Incomplete acidification in meat systems has been directly associated with the accumulation of biogenic amines, including histamine and tyramine, which pose toxicological risks and impair sensory quality [31]. Furthermore, insufficient microbial activity may facilitate the proliferation of spoilage microorganisms and opportunistic pathogens, thereby compromising product safety.

### Fish and seafood fermentation systems

Fermented fish products depend on halophilic and proteolytic microorganisms to develop characteristic flavors and textures [16]. However, ARs present in aquaculture-derived raw materials disrupt these microbial processes. For example, enrofloxacin residues in shrimp have been shown to reduce enzymatic activity and impair flavor development during fish sauce production [45, 46].

Unlike terrestrial systems, aquaculture introduces antibiotics directly into the aquatic environment, resulting in continuous exposure and bioaccumulation that may intensify fermentation disturbances. Consequently, seafood fermentation systems are particularly vulnerable to AR-related technological failures.

### Safety implications of fermentation disruption

The technological risks associated with ARs extend beyond process inefficiency to critical food safety concerns. Fermentation normally lowers pH, creating an environment unfavorable to pathogenic microorganisms. However, when ARs interfere with acidification, pathogens such as *Listeria monocytogenes* and *Salmonella* spp. may survive and proliferate [6, 12]. This disruption not only compromises product safety but also increases the likelihood of foodborne disease outbreaks.

In addition, altered microbial dynamics may favor the emergence and persistence of resistant strains, directly linking technological failure to AMR-related risks.

### Economic and industrial impacts

From an industrial perspective, AR contamination represents a significant economic burden. Fermentation failures result in defective batches that must be discarded, leading to material losses and reduced profitability [11]. Products with compromised quality are less competitive and experience lower market acceptance.

In small-scale and traditional systems, these impacts are often even more severe. For example, contaminated milk in African dairy systems has been associated with reduced viability of locally fermented products, directly affecting farmers' income and food security [42]. On a broader scale, AR contamination may also result in trade restrictions and reputational damage, particularly in export-oriented sectors such as aquaculture [46].

### Limitations of processing interventions

Attempts to mitigate ARs during processing remain largely ineffective. Heat treatment may reduce the concentrations of certain antibiotics; however, several classes, including tetracyclines and sulfonamides, exhibit considerable thermal stability, enabling them to persist during processing and continue exerting inhibitory effects on fermentation microbiota [6].

Consequently, ARs may remain throughout the fermentation process, continuously suppressing microbial activity [31]. This highlights a major limitation of current mitigation approaches: technological interventions applied during processing cannot fully compensate for upstream contamination, emphasizing the need for preventive strategies at the production stage.

### Critical synthesis

Collectively, available evidence demonstrates that ARs constitute a multifaceted technological risk affecting fermentation performance, product quality, food safety, and economic viability. Nevertheless, despite growing recognition of these impacts, current research remains fragmented, with limited integration of microbiological, pharmacological, and technological perspectives.

In particular, the absence of standardized thresholds for fermentation inhibition, the lack of comparative analyses among different fermentation systems, and insufficient attention to artisanal production settings represent major gaps in the literature. Furthermore, limited information is available regarding the long-term effects of low-level AR exposure on fermentation microbiomes and process stability.

Overall, ARs undermine the reliability and sustainability of fermentation-based food production systems. Addressing these challenges requires a comprehensive understanding of their mechanisms of action and technological consequences, which is essential for developing effective mitigation strategies, improving risk assessment, and ensuring the integrity and quality of fermented foods.

## DETECTION AND MONITORING OF ARS

The detection and monitoring of ARs in foods are essential for safeguarding public health, ensuring regulatory compliance, and maintaining the technological integrity of food-processing systems [31]. Antibiotics used in animal production frequently persist in food matrixes such as milk, meat, and fish, necessitating reliable analytical approaches for their identification and quantification [12]. Beyond simple detection, effective monitoring systems must integrate analytical performance, sampling strategies, validation protocols, and surveillance capacity, particularly across diverse regulatory and economic settings [11]. Accurate detection and quantification of ARs in food products require the use of reliable analytical techniques with varying levels of sensitivity, specificity, cost, and operational complexity. Table 3 presents a comparative analysis of the major analytical methods currently employed for the detection of ARs in foods, highlighting their principles, target compounds, analytical performance, practical advantages and limitations, as well as their suitability for implementation in low- and middle-income countries [6, 11, 12, 19, 21, 31, 48].

### Chromatographic methods

Analytical techniques such as LC-MS and gas chromatography–mass spectrometry remain the gold standard for AR detection because of their high sensitivity, specificity, and ability to detect multiple residues simultaneously [19]. These methods enable quantification of antibiotics at concentrations well below MRLs, making them indispensable for regulatory compliance [11]. Liquid chromatography–mass spectrometry, in particular, has demonstrated excellent performance in complex matrixes such as milk, meat, and seafood [12].

Despite their analytical robustness, these techniques are associated with high operational costs, require skilled personnel, and entail labor-intensive sample preparation, which limit their widespread application in resource-constrained environments.

### Immunological and rapid screening methods

ELISA is widely used for large-scale screening because of its simplicity, cost-effectiveness, and relatively rapid turnaround time [31]. These assays have been successfully applied to detect tetracyclines and chloramphenicol in dairy and aquaculture products [6]. In addition to ELISA, rapid screening tools such as lateral flow assays and dipstick tests have emerged as practical solutions for on-farm and in-plant monitoring, enabling early detection of contamination before processing.

However, these methods generally exhibit lower sensitivity and specificity than chromatographic techniques and therefore require confirmatory testing [12]. Nevertheless, their affordability and portability make them particularly attractive for routine screening applications.

### Microbiological assays and low-resource approaches

Microbiological assays remain relevant, particularly in low- and middle-income countries, because of their affordability and ease of use [48]. These methods detect ARs by inhibiting the growth of sensitive bacterial strains [31]. However, their inability to identify specific antibiotic compounds, quantify residue concentrations, or distinguish among residue classes limits their reliability for regulatory purposes. Consequently, microbiological assays are better suited for preliminary screening than for definitive analytical confirmation [6].

### Advanced and emerging technologies

Recent technological developments have expanded the analytical landscape for AR detection. High-resolution mass spectrometry (HRMS) provides enhanced accuracy and enables the detection of a broad spectrum of compounds, including emerging contaminants [19]. Biosensors represent another promising innovation, utilizing biological recognition elements such as enzymes and antibodies to provide rapid, real-time detection of specific antibiotics [21].

These systems have been applied successfully for detecting β-lactams and sulfonamides in milk and meat, offering practical solutions for industrial monitoring [12]. Furthermore, the integration of nanotechnology into biosensor platforms has significantly improved sensitivity, enabling the detection of residues at ultra-low concentrations. Nevertheless, challenges related to standardization, validation, and large-scale implementation remain unresolved [19].

### Analytical challenges and validation constraints

Despite considerable technological progress, several analytical challenges persist. Detection methods must account for matrix effects, whereby food components interfere with analytical signals and potentially affect accuracy and reproducibility [11]. Validation parameters such as limit of detection, limit of quantification, recovery rates, and precision are critical for ensuring method reliability. However, these parameters are not consistently standardized across laboratories and regions, thereby limiting the comparability of results.

Sampling strategies also represent a major constraint. Variations in sampling frequency, sample size, and representativeness may result in underestimation or overestimation of residue prevalence, particularly in heterogeneous food systems. Consequently, harmonization of validation procedures and sampling protocols remains an important priority.

### Surveillance and regulatory monitoring

Surveillance systems play a central role in ensuring food safety and regulatory compliance. In regions such as the European Union and the United States, structured monitoring programs based on routine sampling and testing have proven effective in identifying contaminated products and enforcing compliance with MRLs [12].

**Table 3:** Comparative analysis of analytical methods for the detection of antibiotic residues in foods.

**Method**	**Principle**	**Target compounds**	**Sensitivity (LOD)**	**Specificity**	**Cost**	**Turnaround time**	**Advantages**	**Limitations**	**Suitability for low- and** **middle-income countries**	**References**
LC-MS/MS	Chromatographic separation coupled with mass spectrometric detection	Multi-class antibiotics (β-lactams, tetracyclines, sulfonamides, quinolones)	Very low (ng/kg)	Very high	Very high	Long	Highly sensitive; multi-residue detection; high quantitative accuracy	Expensive; complex sample preparation; requires skilled personnel	Low	[11, 12, 19]
GC-MS	Gas-phase separation with mass detection following derivatization	Volatile or derivatized antibiotics	Low (µg/kg)	High	High	Long	High analytical precision; well established method	Requires derivatization; limited applicability	Low	[19]
HRMS	High-resolution mass spectrometry for exact mass detection	Broad spectrum compounds, including unknown and emerging residues	Ultra-low (ng/kg)	Very high	Very high	Long	Detects emerging contaminants with high accuracy	Very expensive; complex data analysis	Very low	[12, 19]
ELISA	Antibody-antigen interaction	Specific antibiotic classes (tetracyclines, chloramphenicol, β-lactams)	Moderate (µg/kg)	Moderate–High	Low–Moderate	Short	Rapid, cost-effective, and suitable for large-scale screening	Cross-reactivity; requires confirmatory testing	High	[6, 31]
Lateral flow and dipstick tests	Immunochromatographic detection	Target-specific antibiotics	Moderate	Moderate	Low	Very short	Rapid, portable, and suitable for field applications	Lower sensitivity; qualitative or semiquantitative	Very high	[11, 12]
Microbiological assays	Growth inhibition of sensitive bacteria	Broad spectrum antibiotics	Low–Moderate	Low	Very low	Moderate	Simple, inexpensive, and requiring minimal equipment	Nonspecific; no quantification; false positives	Very high	[31, 48]
Biosensors (including nanobiosensors)	Biological recognition coupled with signal transduction	Specific antibiotics	Low to very low	High	Moderate	Very short	Real-time detection; portability; high sensitivity	Limited standardization; emerging technology	Moderate	[19, 21]
High-performance liquid chromatography without mass spectrometry	Liquid chromatographic separation	Selected antibiotic classes	Moderate	Moderate	Moderate	Moderate	Widely available and reliable	Lower sensitivity than LC-MS; limited multi-residue detection	Moderate	[12]

LOD = Limit of detection; LC-MS/MS = Liquid chromatography–tandem mass spectrometry; GC-MS = Gas chromatography–mass spectrometry; HRMS = High-resolution mass spectrometry; ELISA = Enzyme-linked immunosorbent assay; ng/kg = Nanograms per kilogram; µg/kg = Micrograms per kilogram.

However, in many low- and middle-income countries, surveillance systems remain fragmented because of limited infrastructure, inadequate funding, and insufficient technical expertise [6, 48]. This disparity contributes to higher prevalence rates of AR contamination and highlights the need for scalable and cost-effective monitoring solutions.

### Regulatory disparities and global challenges

Inconsistent regulatory frameworks further complicate AR monitoring. MRLs vary considerably among regions, leading to differences in safety standards [4, 11]. These inconsistencies create barriers to international trade and hinder the global harmonization of food safety practices. Products considered compliant in one jurisdiction may be rejected in another, emphasizing the importance of coordinated regulatory approaches.

International organizations such as the Codex Alimentarius Commission and the World Health Organization (WHO) have established guidelines to support harmonized monitoring systems [1, 4]. Nevertheless, the effectiveness of these frameworks ultimately depends on their implementation at the national level, which remains uneven across different regions.

### Critical synthesis

Overall, the detection and monitoring of ARs involve balancing analytical performance, cost, and feasibility. Although advanced techniques such as LC-MS and HRMS provide exceptional accuracy, their accessibility remains limited, particularly in resource-constrained settings. Conversely, rapid screening methods offer scalability and affordability but require confirmatory analyses to ensure reliability.

These considerations highlight the need for integrated monitoring systems that combine complementary analytical approaches. Future efforts should focus on improving the affordability of advanced technologies, harmonizing validation protocols, strengthening sampling strategies, and developing context-specific solutions suitable for both industrial and smallholder production systems.

## MITIGATION STRATEGIES FOR REDUCING ARS IN FOOD

Mitigating ARs in foods requires a comprehensive and integrated strategy that addresses the problem at multiple levels, from primary production to final consumption. Effective mitigation should not only target the root causes of contamination but also incorporate monitoring systems and technological interventions to ensure food safety and maintain technological reliability [12, 31]. To facilitate implementation and improve clarity, mitigation strategies can be categorized into three complementary levels: primary prevention, secondary detection, and tertiary technological intervention. A summary of these approaches is presented in Table 4[1, 4, 11, 12, 19, 21, 31, 48].

**Primary prevention:** Responsible antibiotic use and animal husbandry

The most effective approach for reducing ARs is prevention at the source through responsible antibiotic use in animal production. Antibiotics should be restricted to therapeutic applications and should not be routinely used for growth promotion or prophylaxis [1]. Strict adherence to withdrawal periods is essential to ensure that residues are eliminated from animal tissues before products enter the food chain [11].

Improved animal husbandry practices further contribute to reducing antibiotic dependence. Enhanced hygiene, optimized nutrition, and vaccination programs have been shown to reduce infection rates and decrease the need for antibiotic interventions [21, 48]. However, implementation remains challenging in smallholder production systems because of economic constraints, limited access to veterinary services, and inadequate awareness. These limitations highlight the need for context-specific interventions suitable for both industrial and resource-limited settings.

**Secondary detection:** Monitoring and surveillance systems

Monitoring systems play a critical role in identifying contamination and preventing ARs from entering the food supply. Routine sampling and analytical testing facilitate early detection and regulatory enforcement [12]. Advances in analytical technologies, including HRMS and biosensors, have substantially improved detection capabilities [19, 21]. In addition, rapid screening tools enable on-site testing and support real-time decision-making in food production systems [11].

Despite these advancements, surveillance systems remain unevenly distributed worldwide. In low- and middle-income countries, limited infrastructure, high operational costs, and shortages of technical expertise hinder effective monitoring programs, emphasizing the need for affordable and scalable detection technologies [6, 48].

**Table 4:** Hierarchical mitigation strategies for antibiotic residues in food systems.

**Level**	**Strategy**	**Key actions**	**Target stage**	**Stakeholders**	**Advantages**	**Limitations**	**References**
Primary prevention	Responsible antibiotic use	Restrict use to therapeutic purposes; avoid growth promotion; enforce veterinary prescription	Farm level	Farmers, veterinarians, regulators	Reduces residues at the source; most effective long-term solution	Requires behavioral change; weak enforcement in some regions	[1, 11]
Primary prevention	Withdrawal period compliance	Ensure sufficient time for drug elimination before harvesting products	Farm level	Farmers, regulators	Prevents residues from entering the food chain	Poor compliance because of economic pressures or lack of awareness	[11, 12]
Primary prevention	Improved animal husbandry	Hygiene, nutrition, biosecurity, and vaccination programs	Farm level	Farmers, veterinarians	Reduces disease incidence and antibiotic dependence	Requires infrastructure, training, and investment	[21, 48]
Secondary detection	Routine surveillance systems	Systematic sampling and laboratory testing of food products	Pre-market and market	Regulatory agencies	Enables early detection and regulatory enforcement	High operational costs; limited coverage in low- and middle-income countries	[12, 31]
Secondary detection	Rapid screening tools	ELISA, lateral flow tests, and biosensors for on-site monitoring	Farm and processing	Producers, inspectors	Fast, cost-effective, real-time detection	Lower specificity; requires confirmatory testing	[11, 21]
Secondary detection	Advanced analytical methods	LC-MS and HRMS for confirmatory multi-residue detection	Laboratory	Regulatory laboratories, researchers	High sensitivity and specificity	Expensive; requires technical expertise	[19]
Tertiary intervention	Processing technologies	Enzymatic degradation, adsorption, and residue-reduction methods	Processing stage	Food industry	Can reduce residue levels after contamination	Limited efficiency; not applicable to all antibiotics	[12, 31]
Tertiary intervention	Robust starter cultures	Development of residue-tolerant fermentation cultures	Processing stage	Food technologists, industry	Maintains fermentation performance	Potential AMR risk; regulatory and safety concerns	[21, 31]
Regulatory actions	MRL enforcement	Establish and enforce MRLs	National level	Governments, regulators	Ensures compliance and food safety	Weak enforcement in resource-limited settings	[4, 31]
Regulatory actions	International harmonization	Align standards among countries	Global level	WHO, Codex Alimentarius Commission, governments	Facilitates trade and regulatory consistency	Difficult global coordination	[1, 4]
Consumer-level actions	Awareness and education	Inform consumers about AR and AMR risks	Market	Consumers, public health agencies	Promotes safer consumption patterns	Requires effective communication strategies	[1, 11]
Cross-cutting	Traceability systems	Track the origin and quality of raw materials	Entire supply chain	Producers, regulators	Improves transparency and accountability	Implementation costs and data management complexity	[12]

ARs = Antibiotic residues; AMR = Antimicrobial resistance; ELISA = Enzyme-linked immunosorbent assay; HRMS = High-resolution mass spectrometry; LC-MS = Liquid chromatography–mass spectrometry; MRLs = Maximum residue limits; WHO = World Health Organization.

### Tertiary technological interventions

When contamination occurs, technological interventions may help reduce AR concentrations or mitigate their effects. Processing techniques such as enzymatic degradation and adsorption have shown potential for decreasing ARs without significantly affecting product quality [12].

Another promising approach involves the development of residue-tolerant starter cultures capable of maintaining fermentation performance in the presence of low residue concentrations [21, 31]. However, this strategy raises important safety concerns, particularly regarding the potential selection or dissemination of AMR genes within fermentation microbiota. Consequently, the application of such cultures requires rigorous safety assessments and regulatory evaluation.

### Regulatory and policy interventions

Strong regulatory frameworks are essential for controlling antibiotic use and ensuring compliance with MRLs [4]. Governments must enforce regulations through surveillance programs, inspections, and penalties for noncompliance [31]. International collaboration is equally important for harmonizing standards and improving global food safety.

Organizations such as the Codex Alimentarius Commission and WHO provide guidelines that support coordinated monitoring and regulatory practices [1]. Nevertheless, disparities in regulatory capacity and enforcement remain major challenges, particularly in resource-limited settings where surveillance systems are often fragmented or underdeveloped.

### Consumer awareness and market-based approaches

Consumer education plays a crucial role in mitigating AR-related risks. Public awareness campaigns can improve understanding of AMR and promote responsible consumption behaviors [1, 11]. Market-based mechanisms, including labeling schemes for antibiotic-free products, can provide economic incentives for producers to adopt safer practices [12].

However, the effectiveness of these approaches depends on consumer confidence, regulatory oversight, and the availability of reliable certification systems.

### Sector-specific mitigation strategies

Mitigation approaches should also be tailored to specific production systems. In dairy industries, strict milk screening and appropriate starter culture management are essential. In fermented meat systems, controlling raw material quality and maintaining adequate acidification are critical. In aquaculture, reducing antibiotic use and improving water management practices represent key priorities.

For artisanal and traditional systems, education programs, simplified testing tools, and community-based interventions are particularly important. From an industrial perspective, practical implementation should include systematic screening of raw materials before processing, integration of rapid detection tools at critical control points, and optimization of starter culture selection according to resistance profiles.

Routine screening of milk batches in dairy plants is essential to prevent downstream fermentation failure. In meat fermentation systems, strict control of acidification kinetics may partially offset the inhibitory effects of residues. Similarly, in aquaculture-derived products, pre-processing residue monitoring is crucial for maintaining microbial activity and ensuring product quality.

### Economic considerations and implementation challenges

Implementation of mitigation strategies involves considerable economic considerations. Advanced analytical technologies and improved husbandry practices require substantial investments, which may be prohibitive for small-scale producers. Therefore, cost-benefit analyses and financial support mechanisms are necessary to facilitate adoption, particularly in low- and middle-income countries.

### Critical synthesis

Mitigating ARs in food systems requires a coordinated, multilevel approach integrating prevention, monitoring, technological interventions, and regulatory enforcement. No single strategy is sufficient on its own; instead, combinations of approaches tailored to local conditions are required to ensure effectiveness.

Future efforts should focus on improving the accessibility of technologies, strengthening regulatory frameworks, and developing sustainable and economically viable solutions. In addition, greater emphasis should be placed on the integration of digital surveillance systems, traceability technologies, and risk-based monitoring programs to improve early detection and facilitate coordinated responses.

By integrating these complementary strategies, stakeholders can reduce the prevalence of ARs, protect public health, and preserve the technological integrity and sustainability of fermentation-based food systems [11].

## FUTURE PERSPECTIVES AND RESEARCH DIRECTIONS

The challenges posed by ARs in food systems, particularly in fermentation-based products, necessitate innovative, multidisciplinary, and scalable solutions. Although several mitigation strategies have been proposed, their feasibility, accessibility, and long-term sustainability remain uncertain, particularly across diverse economic and regulatory settings [12, 31]. To address these limitations, future research and policy initiatives should adopt a structured roadmap encompassing short-, medium-, and long-term priorities while explicitly addressing critical knowledge gaps and technological barriers.

**Short-term priorities:** Improving detection and surveillance

In the short-term, strengthening detection and monitoring systems remains a major priority. Advanced analytical technologies such as LC-MS, HRMS, and biosensor platforms offer high sensitivity and specificity for AR detection [19, 21]. However, their widespread implementation is constrained by high costs and technical complexity, particularly in low- and middle-income countries [11, 45].

Future efforts should therefore focus on the development of miniaturized, cost-effective, and user-friendly detection technologies, including portable biosensors and rapid diagnostic kits suitable for field applications. In addition, strengthening surveillance infrastructure and promoting harmonized sampling protocols will be essential for improving the reliability and comparability of monitoring programs.

**Medium-term priorities:** Technological and biological innovations

At the processing level, one promising approach is the development of residue-tolerant or robust starter cultures capable of maintaining fermentation performance in the presence of low concentrations of ARs [12, 31]. Nevertheless, this strategy requires careful evaluation because it may contribute to the selection or dissemination of AMR genes within fermentation ecosystems. Therefore, comprehensive risk assessments involving genomic and phenotypic analyses are essential before large-scale implementation.

Alternative approaches, including probiotics, prebiotics, and phytogenic compounds, have shown promise for reducing antibiotic use at the production stage [6, 12]. However, their scalability, long-term efficacy, and regulatory acceptance remain inadequately characterized, highlighting the need for longitudinal studies and validation across multiple production systems.

**Long-term priorities:** Integration of emerging technologies

In the long-term, emerging technologies are expected to revolutionize AR monitoring and control. Artificial intelligence (AI) and machine-learning approaches can be incorporated into predictive models for contamination risk assessment and fermentation process monitoring. Likewise, metagenomic approaches offer powerful tools for characterizing microbial communities and tracking the resistome in fermented food systems, thereby providing deeper insights into AR–microbiome interactions.

Furthermore, nanotechnology-based biosensors and CRISPR-based detection systems represent promising innovations for the ultrasensitive and rapid detection of ARs. However, successful adoption of these technologies will require standardization, validation, and cost reduction to ensure accessibility across diverse production systems.

### Regulatory harmonization and global governance

Regulatory inconsistencies remain a major obstacle to effective AR control. MRLs vary considerably among countries, creating challenges for international trade and food safety enforcement [1, 4]. Future efforts should prioritize the harmonization of international standards under frameworks such as Codex Alimentarius, accompanied by improved data sharing and coordinated surveillance systems.

Strengthening regulatory capacity in resource-limited settings is particularly important for achieving global consistency. Enhanced international cooperation among governments, scientific institutions, and regulatory agencies will be essential for establishing effective and sustainable control measures.

### Consumer engagement and market transformation

Consumer awareness and behavior are expected to play increasingly important roles in shaping future food systems. As awareness of AMR continues to increase, consumer demand for antibiotic-free products is likely to expand [1, 11]. Future communication strategies should extend beyond health-related concerns and also emphasize technological implications, including fermentation failure and deterioration of product quality, to improve consumer understanding and engagement.

In addition, transparent labeling systems and certification programs may promote market transformation by encouraging producers to adopt safer and more sustainable practices.

## KEY RESEARCH GAPS

Despite increasing interest in ARs, several important knowledge gaps remain:

1. Lack of standardized dose-response thresholds linking AR concentrations with fermentation inhibition.

2. Limited longitudinal studies evaluating the effects of ARs on fermentation microbiomes.

3. Insufficient information regarding the co-occurrence of ARs and AMR genes in fermented foods.

4. Limited integration of advanced analytical tools, including AI and metagenomics, into routine monitoring systems.

5. Scarcity of scalable and economically viable solutions suitable for artisanal and smallholder production systems.

6. Inadequate comparative studies among dairy, meat, and aquatic fermentation systems.

7. Limited understanding of the long-term ecological consequences of chronic low-level AR exposure on fermentation microbiota and food quality.

## STRATEGIC OUTLOOK

Overall, future responses to ARs should avoid reliance on isolated solutions. Instead, an integrated framework combining technological innovation, preventive veterinary practices, accessible monitoring tools, and harmonized regulatory systems is required [11, 31]. Such a framework must also account for economic constraints, regional disparities, and consumer behavior to ensure effective and sustainable implementation.

A One Health-oriented approach integrating veterinary medicine, food technology, microbiology, public health, and regulatory science will be essential for addressing the multifaceted challenges associated with ARs. Only through coordinated, multidisciplinary, and context-specific strategies can ARs be effectively controlled, thereby safeguarding food safety, preserving fermentation technologies, and ensuring the long-term sustainability of global food systems.

## CONCLUSION

ARs in foods of animal origin represent a complex challenge that extends beyond their well established public health implications and directly affects the technological performance and sustainability of fermentation-based food systems. The evidence synthesized in this review demonstrates that ARs are widely distributed across dairy, meat, egg, and aquaculture products, with contamination levels varying according to geographical region, production system, and regulatory capacity. Different antibiotic classes, particularly β-lactams, tetracyclines, sulfonamides, fluoroquinolones, and chloramphenicol, interfere with microbial metabolism and impair key fermentation processes, resulting in delayed acidification, reduced proteolysis, altered flavor development, fermentation failure, and increased risks of pathogen survival and biogenic amine accumulation. The review further highlights the strengths and limitations of currently available analytical approaches and emphasizes the importance of integrating preventive, monitoring, and technological interventions to minimize the occurrence and consequences of AR contamination.

From a practical perspective, effective management of ARs requires responsible antibiotic use, strict compliance with withdrawal periods, improved animal husbandry practices, strengthened surveillance systems, and implementation of rapid and reliable detection technologies. In addition, the development of residue-tolerant starter cultures, innovative biosensor platforms, and digital monitoring tools may provide valuable opportunities for improving fermentation reliability and food safety. Harmonization of international regulatory frameworks and enhancement of monitoring capacities, particularly in low- and middle-income countries, are equally essential for reducing contamination and facilitating safe global trade.

A major strength of this review lies in its integrative approach, which combines microbiological, pharmacological, technological, and regulatory perspectives to provide a comprehensive understanding of the mechanisms and consequences of AR contamination in fermented foods. Unlike previous studies that have focused predominantly on toxicological aspects and AMR, the present review emphasizes the technological dimension of ARs and highlights their implications for fermentation performance and product quality across different food sectors.

Nevertheless, several limitations should be acknowledged. Available evidence remains fragmented, and differences in study design, analytical methodologies, and surveillance capacities limit direct comparisons among studies. Furthermore, standardized dose-response thresholds for fermentation inhibition are lacking, and information regarding the long-term effects of low-level AR exposure on food microbiomes and fermentation ecosystems remains limited. In addition, relatively few studies have systematically compared industrial, artisanal, and traditional fermentation systems.

Future research should prioritize the establishment of standardized fermentation inhibition thresholds, long-term investigations of AR–microbiome interactions, and comparative studies across diverse fermentation systems. Greater integration of advanced technologies, including AI, metagenomics, nanobiosensors, and CRISPR-based detection platforms, may substantially improve monitoring and risk assessment. Further studies are also required to develop scalable and economically sustainable mitigation strategies applicable to both industrial and smallholder production systems.

Overall, ARs should be recognized not only as contaminants of public health concern but also as important determinants of technological performance in fermented foods. Addressing this challenge requires coordinated, multidisciplinary, and One Health-oriented approaches integrating veterinary medicine, food microbiology, analytical sciences, food technology, and regulatory policy. Such efforts will be essential for safeguarding food quality and safety, preserving fermentation technologies, and ensuring the long-term sustainability and resilience of global food systems.

## DATA AVAILABILITY

All data supporting the findings and conclusions of this review are included within the manuscript. 

## GENERATIVE AI DECLARATION

The authors declare that generative artificial intelligence (AI) tools were used solely to improve language, grammar, and readability during manuscript preparation. All scientific content and conclusions were developed and verified by the authors. The authors take full responsibility for the accuracy, integrity, and originality of the work presented, and no AI tool was listed as an author.

## AUTHORS’ CONTRIBUTIONS

MMJA: Conceived and designed the study, coordinated the project, conducted the literature search, analyzed the data, and drafted the original manuscript. BZC, PK, IK, AKLD, and NBAT: Contributed to literature collection, data interpretation, and manuscript revision. EV, NS, MM, NZ, and MA: Participated in critical review, scientific validation, and editing of the manuscript. All authors have read, reviewed, and approved the final version of the manuscript.
